# Amplicon-based metagenomic association analysis of gut microbiota in relation to egg-laying period and breeds of hens

**DOI:** 10.1186/s12866-023-02857-2

**Published:** 2023-05-18

**Authors:** Xiang-Yu Wang, Jin-Xin Meng, Wei-Xin Ren, He Ma, Gang Liu, Rui Liu, Hong-Li Geng, Quan Zhao, Xiao-Xuan Zhang, Hong-Bo Ni

**Affiliations:** 1 College of Veterinary Medicine, Qingdao Agricultural University, Qingdao, Shandong Province 266109 PR China; 2 College of Animal Science & Veterinary Medicine, Heilongjiang Bayi Agricultural University, Daqing, Heilongjiang Province 163319 PR China; 3 College of Veterinary Medicine, Jilin Agricultural University, Changchun, Jilin Province 130118 PR China

**Keywords:** Laying hens, Gut microbiota, 16S rRNA sequencing, Bioinformatic

## Abstract

**Background:**

The gut microbiota plays an essential role in maintaining gut homeostasis and improving performance, with the composition of microbial communities visibly differing across different laying stages in hens and significantly correlating with egg production. To gain further insights into the association between microbial community characteristics and laying periods in Hy-Line variety brown and Isa brown laying hens, we conducted a 16S rRNA amplicon sequencing survey.

**Results:**

Our result revealed the diversity of bacteria in the early laying period was commonly higher than peak, and in Hy-Line variety brown laying hens were generally higher than Isa brown. Principal coordinate analysis (PCoA) and permutational multivariate analysis of variance (PERMANOVA) revealed that the structure and composition of the gut microbiota of laying hens exhibited significant differences among different groups. Phylum *Firmicutes*, *Bacteroidota*, *Proteobacteria*, and *Fusobacteriota* were found that dominant in the host’s feces. Therein, the abundance of *Fusobacteriota* was higher in the peak period than in the early period, while the abundance of *Cyanobacteria* in the early period was higher in two breeds of hens. Furthermore, random forest based on machine learning showed that there were several distinctly abundant genera, which can be used as potential biomarkers to differentiate the different groups of laying periods and breeds. In addition, the prediction of biological function indicated the existing discrepancy in microbial function among the microbiota of four groups.

**Conclusions:**

Our findings offer new insights into the bacterial diversity and intestinal flora composition of different strains of laying hens during various laying periods, contributing significantly to the improvement of production performance and the prevention of chicken diseases.

**Supplementary Information:**

The online version contains supplementary material available at 10.1186/s12866-023-02857-2.

## Background

In humans and animals, every exposed surface (such as skin, mouth, vagina and gut) is colonized by a variety of microorganisms from birth, especially in the gut. These microbes, knows as the microbiota, have been extensively explored in recent years due to their profound implications for host health and productivity [1, 2]. Gut microbiota, as a major regulator of gut function, plays an important role in regulating biological processes associated with nutrient absorption and homeostatic maintenance [3, 4]. Additionally, gut microbiota has immunoregulatory function and is related to certain diseases and detoxification effects [4]. No matter what kind of the functions, it is dependent on the gut microbiota that exist in the gut [5, 6].

More than 1,000 kinds of microbes are found in chicken gut, and play a major role in maintaining the intestinal health and affecting the overall performance of chickens [7]. In recent years, 16S rRNA sequencing has been widely used in soil, plants, marine and gut microbiota due to the rapid development of high-throughput sequencing technology. In a previous study, researchers comprehensively characterized the composition of cecal microbiota of chickens during the whole life cycle, under influence of different breeds, diets and rearing methods [8]. Therein, age was the strongest influencing factor follow by rearing way and breeds. The cecal microbiota of free-range chickens had more diverse and complex and breed had not significant effect on the microbiota of chickens. In addition, Pandit et al. believed that both geographic location and breed have significant effects on composition of cecal microbiota [9]. Studies also have shown that laying stages was a vital factor to change composition of gut microbiota [10]. However, little is known of composition and diversity of gut microbiota of Hy-Line variety brown and Isa brown hens in early and peak laying period.

In this study, we characterized the microbial communities by using 16S V3-V4 region sequencing of amplicon libraries that targeted to bacteria, to thoroughly decipher the composition and diversity of gut microbiota of laying hens, which representing two laying periods and two breeds. Moreover, biomarkers were found by machine learning method to explore important bacteria in host gut. This study provided theoretical basis for maintaining intestinal health, improving dietary nutrition and performance of laying hens.

## Results

### The diversity of gut microbiota of laying hens

**Fig. 1:**
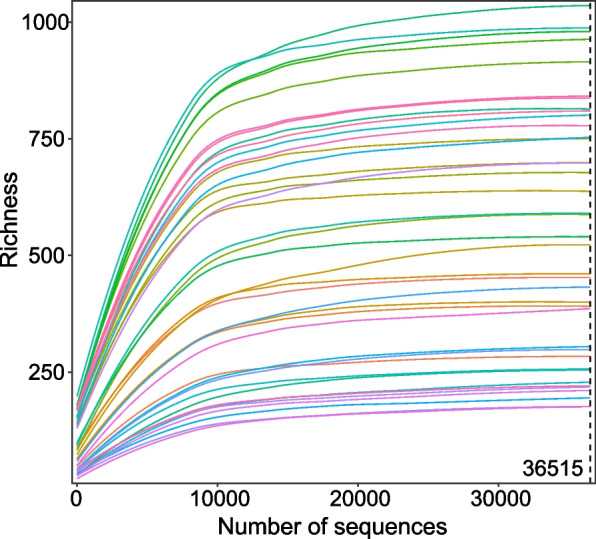
The rarefaction curves tend to attain the saturation plateau showing that the gut microbiota of all samples was large enough to estimate the phenotype richness and microbial community diversity

A total of 3,683,836 raw reads we have acquired after 16S rRNA sequencing of 40 samples, and the datasets were then subjected to quality filtration procedures, which resulted in 3,410,965 clean reads for the subsequent analysis. The average number of sequences per sample was 92,096, and a total of 7270 amplicon sequence variants (ASVs) were identified in the gut bacterial community of laying hens (Supplementary Table 1). Of the 7270 bacterial ASVs observed across all samples, 7161 (98.50%) were identified to phylum, 7157 (98.45%) to class, 7114 (97.85%) to order, 6878 (94.61%) to family and 6148 (84.57%) to genus (Supplementary Table 2). The rarefaction curve, produced by R software, tended to attain the saturation plateau, showing the microbiota of the 40 samples that were large enough to estimate the phenotype richness and microbial community diversity (Fig. 1). Therefore, the results showed that the sequencing data of this experiment are reasonable and accurate.

**Fig. 2:**
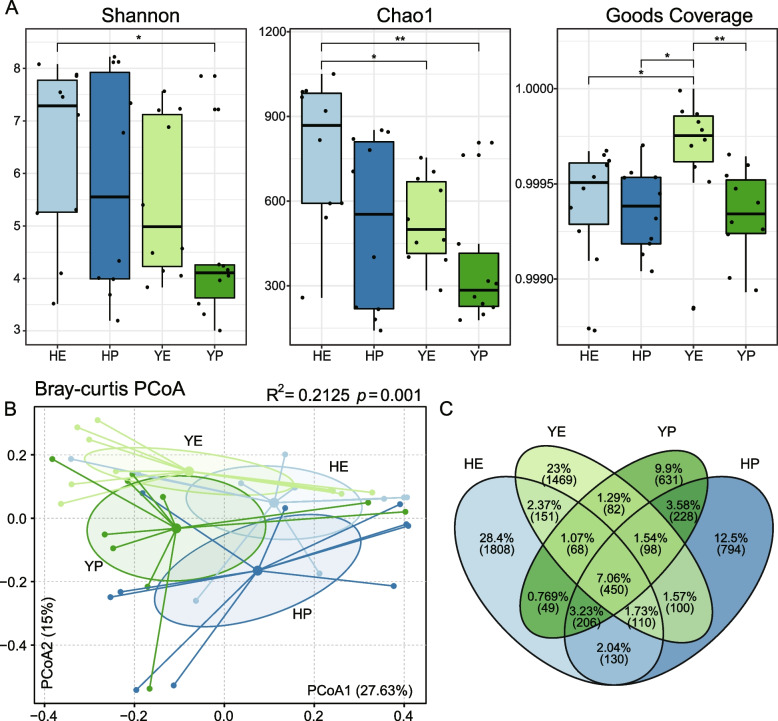
**A** Alpha diversity of two breeds of hens in two laying periods. Wilcoxon rank-sum test: *, *p* < 0.05; **, *p* < 0.01, ***, *p* < 0.001; ****, *p* < 0.0001. **B** Principal coordinate analyses (PCoA) and permutation multiple variance analysis (PERMANOVA) show the structural differences in the communities of gut bacteria. **C** Distribution of amplicon sequence variants (ASVs) across different groups

In order to measure the α-diversity of microbiota in the gut of laying hens, indices for Shannon, Chao1 and goods coverage were calculated. Interestingly, three indices in early laying period were commonly higher than in peak, although there were not statistically different except index for Goods Coverages between groups YE and YP (*p* < 0.01, Fig. 2A). Similarly, three indices for Hy-Line variety brown laying hens were generally higher than Isa brown except Goods Coverage index in early laying period. Among these, Chao1 index for group HE was observed that significantly (*p* < 0.05) higher than YE, while Goods Coverage for group HE was significantly (*p* < 0.05) lower than YE.

To visualize the structural characteristic in the gut bacterial communities among different groups, the principal coordinate analysis (PCoA) based on the Bray–Curtis distances was performed. The PCoA result indicated that the PCoA axes 1 and 2 accounted for 27.63% and 15.00% of the total variation, respectively. And four groups formed clusters with a partial overlap were observed in plot (Fig. 2B). Further analysis using permutation multiple variance analysis (PERMANOVA) showed that the gut microbiota composition of the laying hens exhibited significant difference among different groups (R^2^ = 0.2125, *p* = 0.001).

### Variation in gut microbiota structure of laying hens

**Fig. 3:**
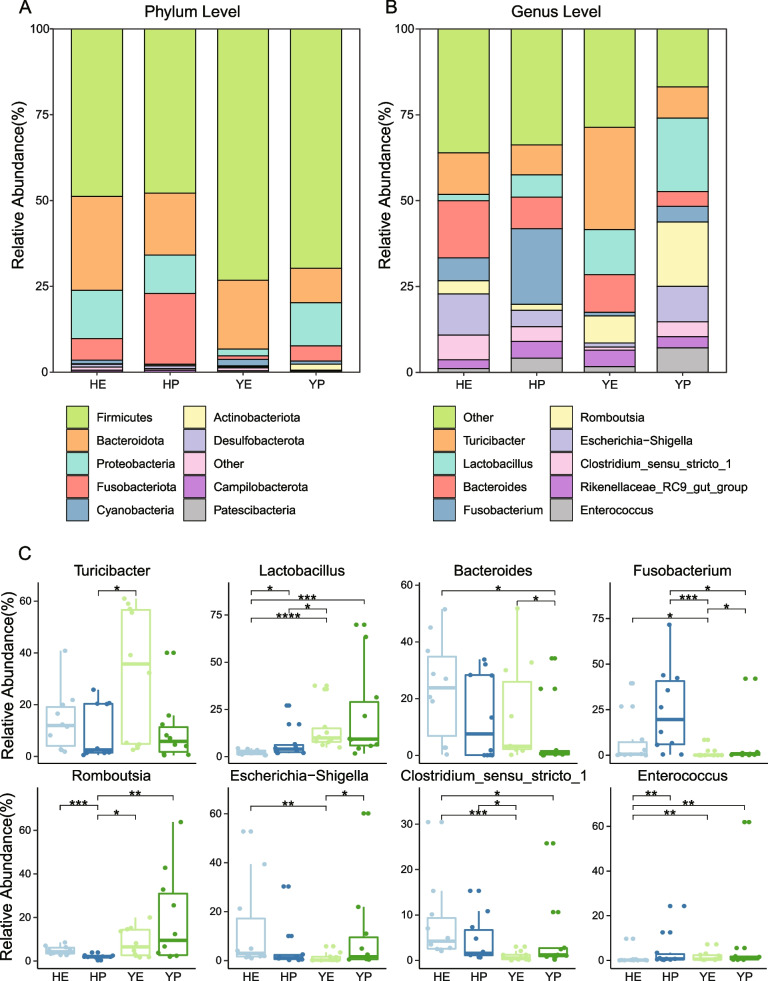
**A**, **B** Community composition of the gut microbiota among four groups of laying hens at the phylum and genus levels, respectively. **C** The eight genera existing significantly difference in the feces of laying hens. Wilcoxon rank-sum test: *, *p* < 0.05; **, *p* < 0.01, ***, *p* < 0.001; ****, *p* < 0.0001

The gut microbiota composition of laying hens in four groups showed a marked variation in the relative abundance of taxa. In phylum level, all sequences were classified into 30 phyla, although only 4 phyla were most common (average relative abundance > 1%), including *Firmicutes* (47.86–73.22%), *Bacteroidota* (10.11–27.34%), *Proteobacteria* (1.97–14.06%) and *Fusobacteriota* (1.03–20.62%, Fig. 3A, Supplementary Table 3). In the feces of Hy-Line variety brown laying hens, *Fusobacteriota* was significantly (*p* < 0.05) less in HE than HP, while *Cyanobacteria* was significantly (*p* < 0.01) higher in HE than HP (Fig. S1A). Moreover, compared with Isa brown laying hens, *Bacteroidota*, *Cyanobacteria*, *Patescibacteria* and *Euryarchaeota*, in top 10 of relative abundance, were significantly (*p* < 0.05) higher in YE than YP. Nonetheless, *Proteobacteria* and *Fusobacteriota* were prominently (*p* < 0.05) higher in YP than YE. Intriguingly, we discovered that *Fusobacteriota* was commonly higher in peak laying hens than early, but the opposite is true in *Cyanobacteria*. Furthermore, *Firmicutes* was significantly (*p* < 0.05) less in Hy-Line variety brown laying hens than Isa brown, while *Fusobacteriota* was significantly (*p* < 0.05) more in Hy-Line variety brown than Isa brown.

In genus level, a total of 514 genera were identified in 40 fecal samples, and the top 5 for average relative abundance were *Turicibacter* (8.69–29.83%), *Lactobacillus* (1.83–21.33%), *Bacteroides* (4.31–16.67%), *Fusobacterium* (1.08–22.06%) and *Romboutsia* (1.72–18.73%, Fig. 3B). In Hy-Line variety brown laying hens, *Lactobacillus* and *Enterococcus* were obviously dominated in HP, while *Romboutsia* was enriched in HE (*p* < 0.05, Fig. 3C). In Isa brown, *Fusobacterium* and *Escherichia-Shigella* were significantly (*p* < 0.05) higher inYP than YE, but *Bacteroides* was higher in YE.

### Unique, shared and core ASVs in laying hens of four groups

To investigate the distribution of gut microbiota in different groups, the analysis of common, unique and core ASVs was conducted, as shown in the Venn diagram (Fig. 2C). The unique ASVs in HE were the most numerous, which accounted 28.4% (1808), followed by group YE (1469, 23.0%), HP (794, 12.5%) and YP (631, 9.9%). The Hy-Line variety brown hens from two different groups were shared 130 ASVs, Isa brown hens shared 82. Excluded the influence of breeds, 228 ASVs were shared between peak laying periods and 151 ASVs were shared between early laying periods. The concept of “core microbiota” is used to identify and describe key microorganisms that are stable and permanent in a microbial community [11]. Here, the core ASVs were defined as bacteria that existed in each group. Therefore, 450 ASVs were shared in all groups, which mostly belong to phylum *Firmicutes* (365) and *Bacteroidota* (48) or family *Ruminococcaceae* (68), *Lachnospiraceae* (60) and *Oscillospiraceae* (56).

### Gut microbiota as biomarkers for different varieties and egg laying periods

**Fig. 4:**
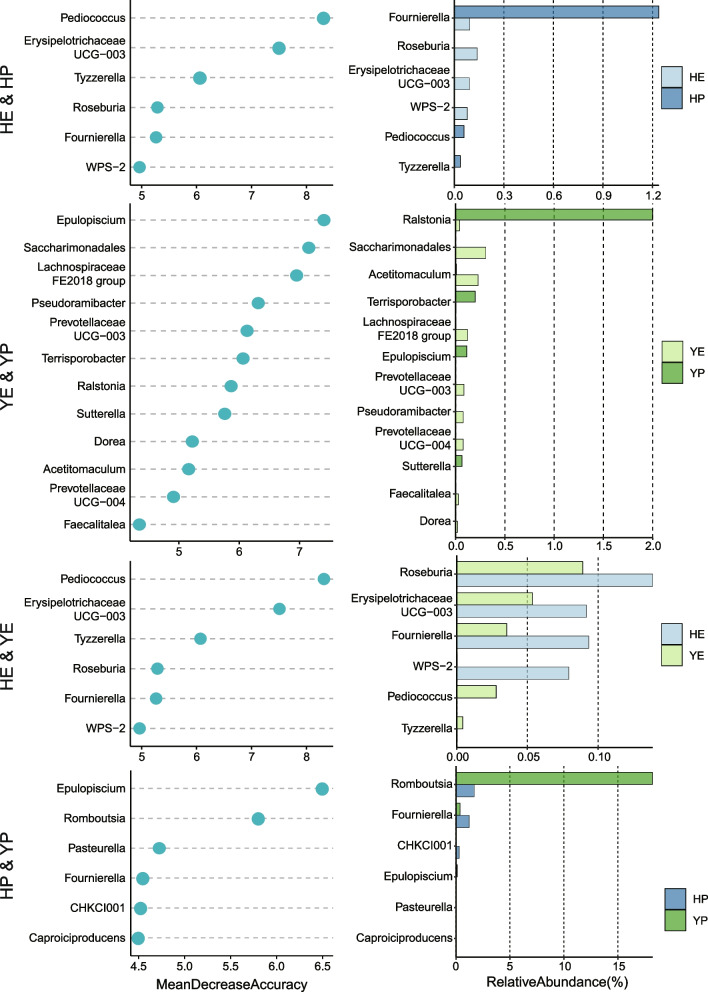
Random forest based on machine learning to explore biomarker of genera between each group pair. HE versus HP, YE versus YP, HE versus YE, HP versus YP. Bar plot showed relative abundance of biomarkers in groups

To discovered whether members of gut bacteria can be used as biomarkers to differentiate laying period or breeds, here we established models using the machine-learning random forest approach to correlate laying periods and breeds of laying hens with genus-level gut microbiota data. We carried out five-fold cross-validation with five repeats to evaluate the importance of indicator bacterial genera (Fig. S1B). The method is referred to another passage, which has been recognized and applied by the peers [12]. Thus, we defined the top 12 genera as biomarkers in the model for YE&YP, and top 6 were defined in models for other group pairs, in order of group-discriminatory importance (MeanDecreaseAccuracy, MDA), respectively (Fig. 4). As the Fig. 4 shown, *Pediococcus*, *Erysipelotrichaceae UCG-003*, *Tyzzerella*, *Roseburia*, *Fournierella* and *WPS-2* were the most important genera to discriminate laying periods between HE and HP,, while *Epulopisciu*, *Saccharimonadales* etc. were vital biomarkers to differentiate between YE and YP. Moreover, *Pediococcu*, *Erysipelotrichaceae UCG-003*, *Tyzzerella*, *Roseburia*, *Fournierella* and *WPS-2* were used to differentiate hens of two breeds in early laying period, while *Epulopiscium*, *Romboutsia*, *Pasteurella*, *Fournierella*, *CHKCI001* and *Caproiciproducens* were as biomarkers to differentiate in peak. In addition, we employed the bar plot to display the relative abundance of bacterial biomarkers.

### Functional predictions of gut microbiota laying hens

**Fig. 5:**
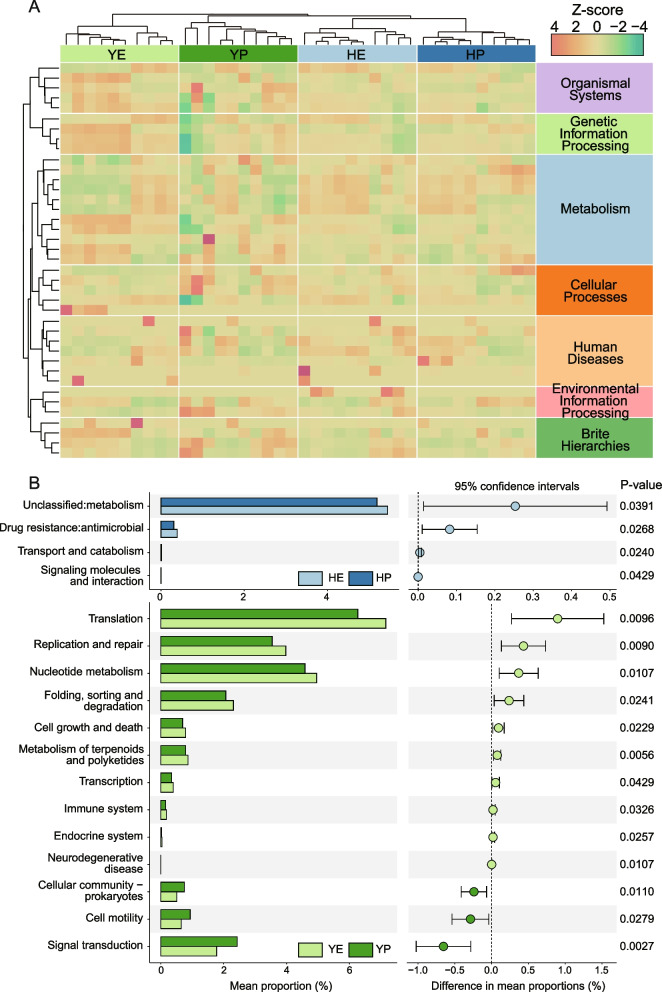
**A** KEGG metagenomic function of bacteria was predicted using the PICRUSt2 pipeline. The heatmap display abundance of function at KEGG level B. The value represented the normalization of functional abundance in this dataset, which higher numbers indicated greater relative abundances with colors ranging from dark red to green. **B** Several functions were detected existing significant difference in HE and HP as well as YE and YP

For further understanding of the biological function of the microbial community, the metagenomic functions of bacteria were predicted by employing PICRUSt2 pipeline. There were 7262 predicted metagenomic functions obtained and annotated using KEGG Orthology (KO, Supplementary Table 4). 2574 KO were found for metabolism, genetic information processing, environmental information processing, cellular processes and etc. According to the hierarchical relationship of pathways, all KO were classified as pathway of level B in order to descript and compare function in gut microbiota of laying hens. Various degrees of functional pathways of microbiota were observed in different groups as shown in the heatmap (Fig. 5A), suggesting a discrepant microbial functional potential among microbiota of several groups. 4 and 13 pathways were considered to have significant (*p* < 0.05) differences between early and peak laying period in both two breeds, respectively (Fig. 5B). Antimicrobial drug resistance, transport and catabolism as well as signaling molecules and interaction were had a significantly higher abundance in HE compared to HP. 9 pathways were shown to be the most abundance function in YE, including translation, replication and repair, nucleotide metabolism, metabolism of terpenoids and polyketides and etc., but functions of signal transduction, cell motility and prokaryotes of cellular community had a preference for YP.

## Discussion

Chickens represent one of the most widespread farm animals worldwide, and their egg is also important source of animal-protein for humans [13]. The microbiota, colonizing in the chicken gut, plays a vital role in maintaining gut health and influences the overall performance. Thus, a better understanding of the structure and diversity of gut microbiota will facilitate managing microbial community of laying hens to achieve better health and productivity. In our study, we explored the differences of bacterial composition and diversity pattern of the Hy-Line variety brown and Isa brown laying hens in two different laying periods and further to detect important bacteria which could as biomarkers to discriminate different groups. Additionally, metagenomic function of laying hens were predicted to compare functional difference between groups. The study suggested that factor of laying periods and breeds exert a stronger determinant of the composition and diversity of the hens than individual differences.

We calculated indices for Shannon, Chao1, and goods coverage to evaluate α-diversity of microbiota in hen’s gut. Indices in early laying period were commonly higher than in peak, although not all significant. There was evidence that higher levels of richness and diversity of gut microbiota are correlated with positive health outcomes [14]. A previous study showed that gut microbiota had higher richness and diversity in peak than early laying period [10], and another study reported richness was increased with age from hatching to end of lay [15]. However, some studies have found the reverse, with better performance correlated with lower richness and diversity in feces [16]. A potential reason may be contributed to phyla *Proteobacteria* and *Actinobacteria* enriched in the peak period (Fig. S1A, ST3), these phyla consist of pathogenic microbes that disrupt the microbiota composition and overwhelm the intestinal homeostasis by producing toxins and harmful agents [17–19]. Additionally, stress response and management during feeding as well as chicken breeds were also underlying factors. In beta diversity, significant difference among different groups was detected, although existing a certain overlap between clusters. Some similarities in the composition of the gut microbiota maybe because same chickens were followed-up at early and peak period.

In our study, we identified microbes belonging to four groups at phylum and genus levels of taxa, and evaluated their abundance to provide detailed information regarding the composition of the fecal microbiota. Therein, as reported in previous studies in other chicken breeds [10, 15, 19, 20], *Firmicutes*, *Bacteroidota*, *Proteobacteria* and *Fusobacteriota* were dominant phyla in all laying hens, and accounted at least 96% of total abundance in fecal samples (Fig. 3, Supplementary Table 3). Actually, the high ratio between *Firmicutes* and *Bacteroidetes* was estimated in the peak laying period of hens, in order to promoting intestinal absorption and energy biosynthesis, and its functionality has been reported in other various species [21, 22]. A higher ratio of F/B in the fecal microbiota contributed to the enhancement of utilization efficiency of feed energy [19, 23]. Additionally, we found *Fusobacteriota* had higher abundance in peak than early period of two chicken breeds, but *Cyanobacteria* was highly enriched in early, these characters were also as similar as other report about Jing Hong and other commercial hens [19]. Interestingly, Hy-Line variety brown hens was similar to Jing Hong chicken which both had higher abundance of *Proteobacteria* in early period, but the opposite is true in Isa brown may be attributable to Isa brown breed had a major influence on it. In genus level, we discovered that *Lactobacillus* was enriched in peak period group, furthermore, Isa brown had higher abundance than Hy-Line variety brown hens. There was a study showed that adverse effects of *Lactobacillus* on weight gain in the broiler chickens, however, several studies also indicated that *Lactobacillus* had positive effects to improve gut health and productive performance [24, 25]. Thus, our result seemed to support the latter view, although still there were controversies regarding the role of *Lactobacillus* in chickens. In addition to that a previous study indicated that *Turicibacter* showed a negative correlation with egg weight and laying rate of laying hens [26]. In line with these findings, *Turicibacter* was notably less in peak period than early. Indeed, different breeds, in other words, different genotype had a visible influence on structure of gut microbiota. For example, a paper indicated that genotypes can have a significant impact on the composition of the intestinal microbiota, resulting in significantly difference of microbiota between different breeds [27]. Consequently, our result about gut microbiota of different breeds was consistent with this view.

In this study, classified algorithm of random forest was used to explore the gut microbial marker of laying hens between groups. *Pediococcus*, *Tyzzerella, Roseburia* and other three genera were defined as biomarkers between early and peak laying period of Hy-Line variety brown hens (Fig. 4). Several previously published studies reported that *Pediococcus*, which had probiotics properties, were competitively inhibit the growth of *Salmonella* and control the inflammatory response in chicken gut [28, 29]. *Pediococcus* was highly enriched in peak period, which beneficial to gut health and performance of improvement. And *Epulopiscium*, *Saccharimonadales*, *Lachnospiraceae FE2018 group* and other 9 genera were identified as biomarkers of Isa brown hens. Furthermore, 6 biomarkers for HE&YE and 6 for HP&YP were also identified to differentiate different groups.

A total of 7262 predicted biological functions were obtained and converted to level B of KEGG pathway. As shown as heatmap, in the different group, the functional profiles of the gut microbiota changed (Fig. 5A). Meanwhile, function such as antimicrobial of drug resistance, transport and catabolism as well as signaling molecules and interaction were significant enriched in HE compared to HP, and 12 pathways between YE and YP had significantly difference. Several metabolic pathways were differentially enriched between Hy-Line variety brownand Isa brown hens in current study, consistent with a previous study, which were further dependent on laying hen strains [30]. Actually, the laying periods transition from early to peak, as the way of growing in age, metabolism had experienced a complicated changed [31]. Nonetheless, only several metabolism-related functions were observed that exiting notably difference between early and peak period. Therein, signal transduction, cell motility and prokaryotes of cellular community were found a preference for YP (Fig. 5B). Another research was also reported the similar findings in Ninghai Indigenous Chickens in two different laying periods, which proved to be closely correlated to egg production [32]. In addition to that abundance of some pathways was not altered apparently between two periods. This could be attributed to no appreciable difference in phyla *Firmicutes* and *Bacteroidota* between early and peak period, or prediction error, and maybe even fecal sample not reflect the real metabolic state in our study. As such, one needed to pay special attention to rational management and feeding in case disease and reduction in production.

## Conclusion

In summary, increased knowledge of gut microbial community of early and peak laying period in two chicken breeds has been produced, with the goal of providing new insights and improving gut health and performance. In this study, we noted significant difference in gut microbial community among groups, indicating that laying period and breeds have important influence on the diversity and composition of the gut microbiota. Firstly, the gut microbiota in early laying period was more diverse than peak, and in Hy-Line variety brown than Isa brown. Secondly, *Firmicutes*, *Bacteroidota*, *Proteobacteria* and *Fusobacteriota* were dominated in host’s feces. And the high ratio F/B was estimated in the peak laying period of hens. Additionally, we found *Fusobacteriota* had higher abundance in peak than early period of two chicken breeds, but *Cyanobacteria* was highly enriched in early. Therefore, measures of adding probiotics or prebiotics, and diversifying the diets should be considered to relieve reduction of intestinal microbial diversity in peak laying period, which to the benefit of maintaining the stability of intestinal homeostasis and resisting invasion of opportunistic pathogens. Meanwhile, adding some microbial agents based on *Fusobacterium* into the diet of laying hens during the laying period can appropriately increase the laying peak period of laying hens and thus increase the yield. And more surveillance should be taking into account to distinguish some bacteria, belonging to *Proteobacteria* and *Cyanobacteria*, are whether symbiotic partner or harmful pathogens. In the future, it will be interesting to explore the bacteria which significantly different between laying periods or breeds, finding probiotic candidates or pernicious bacteria, to maintain gut health and to enhance production.

## Materials and methods

### Animals details

ISA brown and Hy-Ling brown laying hens raised in cages since birth were selected from a commercial chicken farm in Jimo District, Qingdao City, Shandong Province, China. The laying hens of the same age were under the same breeding system, including management program and diet. In terms of feeding regimen, the farm uses standard and same daily diet at each age and devoid of the antimicrobials or antibiotics. Different laying periods have a slight difference in the diet. During early laying period(120 days old), the feed formula included corn (62.5%), soybean meal (24%), oil(0.5%), stone powder (8%) and premix (5%). During the peak laying period (180 days old), the feed formula included corn (61%), soybean meal (25%), oil(1%), stone powder (8%) and premix (5%). The laying hens did not receive any feed supplements throughout their life cycle.

### Sample collection

The sampling process is detailed as follows. We put on lab clothes and sterile gloves and masks and waited next to the chicken coops. We put clean plastic bags into the chicken coop, and the feces would fall on the plastic bag when the hens excreted. Then the plastic bag would be carefully taken out, and the feces would be put into 50 ml sterile centrifugal tube. Fresh fecal samples were collected, frozen using liquid nitrogen, and transported to the laboratory in a dry-ice pack, then stored at − 80 °C until DNA extraction. A total of 40 samples were collected in two chicken commercial excellent breeds. These included the early laying periods (120 days old, YE1-10) and peak laying periods (180 days old, YP1-10) of Isa brown laying hens; the early laying periods (120 days old, HE1-10) and peak laying periods (180 days old, HP1-10) of Hy-Line variety brown laying hens.

### DNA extraction, 16S rRNA gene amplification and sequencing

DNA extraction was carried out immediately from the collected fecal samples using the TIANGEN stool DNA kit (TIANGEN Biotech Co., Ltd., Beijing, China). PCR amplification of the V3-V4 region of 16S rRNA was using the primers: 341F (5’ CCTACGGGNGGCWGCAG-3’) and 806R (5’ GGACTACHVGGGTWTCTAAT-3’). The reaction of PCR was carried out in the 20 μL system that contained 4 μL 5 × Taq Buffer, 2 μL dNTPs, 0.8 μL of each primer, 0.4 μL Taq DNA Polymerase, 1 μL DNA template, and 11 μL ddH_2_O. PCR reaction conditions are as follows: pre-denaturation at 95 °C for 30 s, and followed by 35 cycles of denaturation at 95 °C for 30 s, annealing at 59 °C for 30 s, and extension at 72 °C for 45 s, and a final extension at 72° C for 10 min. The PCR amplification product was detected by 2% agarose gel electrophoresis, and the sequences with the main band size between 400 and 450 bp were selected. The product purification kit uses the Thermo GeneJET Gel Extraction Kit. Illumina TruSeq DNA PCR-Free Library Preparation Kit library kit was used to construct the library. And the library was quantified by Qubit. Finally, NovaSeq 6000 platform was used for 250 bp paired-end sequencing.

### Bioinformatics

All the raw sequences were filtered for quality control to get operational sequences firstly. The sequences were identified using QIIME 2 software [33], DADA2 was employed to remove the primers, denoise, and join the reads into exact amplicon sequence variants (ASVs) [34]. QIIME2 was used to assign taxonomies with the feature-classifier plugin [35]. Phylogenetic trees were constructed with the FastTree plugin [36]. The original table was flattened for subsequent analysis, and a read depth of 36,515 was set for sample normalization. Alpha and beta diversity were evaluated by the QIME2 pipeline. Indices for Shannon, Goods Coverage and Chao1 were calculated to measure the α-diversity in the QIIME2 pipeline, and calculated the Bray–Curtis distances to measure β-diversity. PICRUSt2 plugin for QIIME2 and KEGG Orthology database were adopt to further predictive functional analysis [37, 38].

### Data analysis

After filtering features with relative abundances less than 0.01% and prevalence rate less than 10%, PCoA and data visualization were performed using vegan (vegan, v2.5–7) and ggpubr (v0.4.0), respectively, to assess the microbiota between different sample groups structural differences. Wilcoxon rank sum test was employed to evaluate difference for alpha diversity index and relative abundance of taxa (phylum and genus level). PERMANOVA (999 permutations) was employed to identify significant differences between groups [39]. Student’s t-test was adopt to test for significance of microbial function between the two groups. We conducted random forest classification model to predict breeds and egg producing periods based on RandomForest package (v4.6–14). Heatmaps were generated in R with the pheatmap (v1.0.12) and ComplexHeatmap packages (v2.8.0). Venn diagrams were generated by VennDiagram (v1.6.20) packages. And other visualizations were based on the ggplot2 package (v3.3.5). All graphical presentations were generated under the R environment (v4.1.1).

## Supplementary Information

**Additional file 1:** **Supplementary Figure 1.** (A) Boxplot show the relative abundance and difference of nine phyla of bacteria among four groups of laying hens. Wilcoxon rank-sum test: *, *P* < 0.05; **, *P* < 0.01, ***, *P* < 0.001; ****, *P* < 0.0001. (B) Five-fold cross-validation with five repeats were used to evaluate the importance of indicator bacterial genera, including HE versus HP, YE versus YP, HE versus YE, HP versus YP.

**Additional file 2:** **Supplemental Table 1.** Number of sequence of ASVs in samples. **Supplemental Table 2.** Taxonomy information of ASVs. **Supplemental Table 3.** Proportion of bacterial abundance of four groups, including top 10 phyla and top 20 genus. **Supplemental Table 4.** The abundance of KO in each sample and KO hierarchy relationship predicted by PICRUSt2.

## Data Availability

The 16S rRNA raw sequence data have been deposited at China National Center for Bioinformation under the accession code PRJCA010671.

## References

[1] Dethlefsen L, McFall-Ngai M, Relman DA (2007). An ecological and evolutionary perspective on human-microbe mutualism and disease. Nature.

[2] Round JL, Mazmanian SK (2009). The gut microbiota shapes intestinal immune responses during health and disease. Nat Rev Immunol.

[3] Qin J, Li R, Raes J, Arumugam M, Burgdorf KS, Manichanh C (2010). A human gut microbial gene catalogue established by metagenomic sequencing. Nature.

[4] Subramanian S, Huq S, Yatsunenko T, Haque R, Mahfuz M, Alam MA (2014). Persistent gut microbiota immaturity in malnourished Bangladeshi children. Nature.

[5] Donaldson RM (1964). Normal bacterial populations of the Intestine and their relation to intestinal function. N Engl J Med.

[6] Cheng LK, O’Grady G, Du P, Egbuji JU, Windsor JA, Pullan AJ (2010). Gastrointestinal system. WIREs Mech Disease.

[7] Khan S, Moore RJ, Stanley D, Chousalkar KK (2020). The gut microbiota of laying hens and its manipulation with prebiotics and probiotics to enhance gut health and food safety. Appl Environ Microbiol.

[8] Ocejo M, Oporto B, Hurtado A (2019). 16S rRNA amplicon sequencing characterization of caecal microbiome composition of broilers and free-range slow-growing chickens throughout their productive lifespan. Sci Rep.

[9] Pandit RJ, Hinsu AT, Patel NV, Koringa PG, Jakhesara SJ, Thakkar JR (2018). Microbial diversity and community composition of caecal microbiota in commercial and indigenous Indian chickens determined using 16s rDNA amplicon sequencing. Microbiome.

[10] Van Goor A, Redweik GAJ, Stromberg ZR, Treadwell CG, Xin H, Mellata M (2020). Microbiome and biological blood marker changes in hens at different laying stages in conventional and cage free housings. Poult Sci.

[11] Astudillo-García C, Bell JJ, Webster NS, Glasl B, Jompa J, Montoya JM (2017). Evaluating the core microbiota in complex communities: A systematic investigation. Environ Microbiol.

[12] Zhang J, Liu YX, Zhang N, Hu B, Jin T, Xu H (2019). NRT1.1B is associated with root microbiota composition and nitrogen use in field-grown rice. Nat Biotechnol.

[13] Bain MM, Nys Y, Dunn IC (2016). Increasing persistency in lay and stabilising egg quality in longer laying cycles. What are the challenges?. Br Poul Sci.

[14] Stanley D, Hughes RJ, Moore RJ (2014). Microbiota of the chicken gastrointestinal tract: influence on health, productivity and disease. Appl Microbiol Biotechnol.

[15] Joat N, Van TTH, Stanley D, Moore RJ, Chousalkar K (2021). Temporal dynamics of gut microbiota in caged laying hens: a field observation from hatching to end of lay. Appl Microbiol Biotechnol.

[16] Siegerstetter S-C, Schmitz-Esser S, Magowan E, Wetzels SU, Zebeli Q, Lawlor PG (2017). Intestinal microbiota profiles associated with low and high residual feed intake in chickens across two geographical locations. PLoS ONE.

[17] Chen Y-J, Wu H, Wu S-D, Lu N, Wang Y-T, Liu H-N (2018). Parasutterella, in association with irritable bowel syndrome and intestinal chronic inflammation. J Gastroenterol Hepatol.

[18] Rizzatti G, Lopetuso LR, Gibiino G, Binda C, Gasbarrini A (2017). Proteobacteria: a common factor in human diseases. Biomed Res Int.

[19] Elokil AA, Magdy M, Melak S, Ishfaq H, Bhuiyan A, Cui L (2020). Faecal microbiome sequences in relation to the egg-laying performance of hens using amplicon-based metagenomic association analysis. Animal.

[20] Su Y, Ge Y, Xu Z, Zhang D, Li D (2021). The digestive and reproductive tract microbiotas and their association with body weight in laying hens. Poult Sci.

[21] Turnbaugh PJ, Ley RE, Mahowald MA, Magrini V, Mardis ER, Gordon JI (2006). An obesity-associated gut microbiome with increased capacity for energy harvest. Nature.

[22] Murphy EF, Cotter PD, Healy S, Marques TM, O’Sullivan O, Fouhy F (2010). Composition and energy harvesting capacity of the gut microbiota: relationship to diet, obesity and time in mouse models. Gut.

[23] Wang Y, Xu L, Sun X, Wan X, Sun G, Jiang R (2020). Characteristics of the fecal microbiota of high- and low-yield hens and effects of fecal microbiota transplantation on egg production performance. Res Vet Sci.

[24] De Cesare A, Sirri F, Manfreda G, Moniaci P, Giardini A, Zampiga M (2017). Effect of dietary supplementation with Lactobacillus acidophilus D2/CSL (CECT 4529) on caecum microbioma and productive performance in broiler chickens. PLoS ONE.

[25] Forte C, Manuali E, Abbate Y, Papa P, Vieceli L, Tentellini M (2018). Dietary Lactobacillus acidophilus positively influences growth performance, gut morphology, and gut microbiology in rurally reared chickens. Poult Sci.

[26] Gan L, Zhao Y, Mahmood T, Guo Y (2020). Effects of dietary vitamins supplementation level on the production performance and intestinal microbiota of aged laying hens. Poult Sci.

[27] Zhao L, Wang G, Siegel P, He C, Wang H, Zhao W (2013). Quantitative genetic background of the host influences gut microbiomes in chickens. Sci Rep.

[28] Torok VA, Ophel-Keller K, Loo M, Hughes RJ (2008). Application of methods for identifying broiler chicken gut bacterial species linked with increased energy metabolism. Appl Environ Microbiol.

[29] Lan D, Xun X, Hu Y, Li N, Yang C, Jiang X (2020). Research on the effect of pediococcus pentosaceus on salmonella enteritidis-infected chicken. Biomed Res Int.

[30] Adhikari B, Jun S-R, Kwon YM, Kiess AS, Adhikari P (2020). Effects of housing types on cecal microbiota of two different strains of laying hens during the late production phase. Front Vet Sci.

[31] Bendikov-Bar I, Malitsky S, Itkin M, Rusal M, Sagi D (2021). Metabolomic changes are predictive of aging in laying hens. J Gerontol: Series A.

[32] Huang X, Zhou W, Cao H, Zhang H, Xiang X, Yin Z (2022). Ovarian transcriptomic analysis of Ninghai indigenous chickens at different egg-laying periods. Genes.

[33] Bolyen E, Rideout JR, Dillon MR, Bokulich NA, Abnet CC, Al-Ghalith GA (2019). Reproducible, interactive, scalable and extensible microbiome data science using QIIME 2. Nat Biotechnol.

[34] Callahan BJ, McMurdie PJ, Rosen MJ, Han AW, Johnson AJA, Holmes SP (2016). DADA2: High-resolution sample inference from Illumina amplicon data. Nat Methods.

[35] Quast C, Pruesse E, Yilmaz P, Gerken J, Schweer T, Yarza P (2012). The SILVA ribosomal RNA gene database project: improved data processing and web-based tools. Nucleic Acids Res.

[36] Price MN, Dehal PS, Arkin AP (2009). FastTree: computing large minimum evolution trees with profiles instead of a distance matrix. Mol Biol Evol.

[37] Douglas GM, Maffei VJ, Zaneveld JR, Yurgel SN, Brown JR, Taylor CM (2020). PICRUSt2 for prediction of metagenome functions. Nat Biotechnol.

[38] KanehIsa brown M, Goto S (2000). KEGG: kyoto encyclopedia of genes and genomes. Nucleic Acids Res.

[39] Li M-H, Meng J-X, Wang W, He M, Zhao Z-Y, Ma N (2022). Dynamic description of temporal changes of gut microbiota in broilers. Poult Sci.

