# Supplementary figures and images for: Amplicon-based metagenomic association analysis of gut microbiota in relation to egg-laying period and breeds of hens
Source: BMC Microbiol. 2023 May 18;23:138. doi: 10.1186/s12866-023-02857-2 (PMC10193603; doi:10.1186/s12866-023-02857-2)

# Supplementary Figure

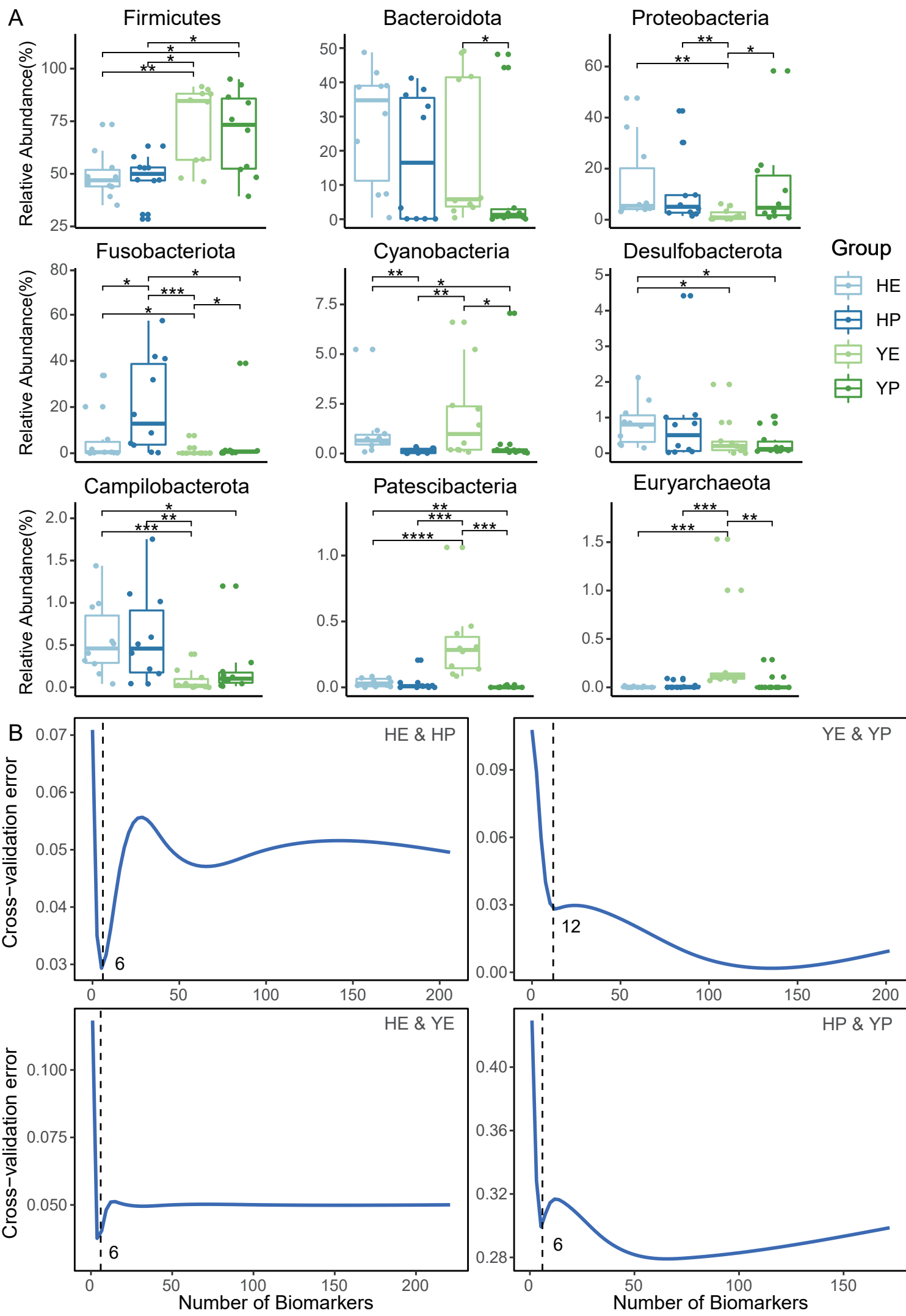

Supplement: Supplementary file 1 — Additional file 1: Supplementary Figure 1. (A) Boxplot show the relative abundance and difference of nine phyla of bacteria among four groups of laying hens. Wilcoxon rank-sum test: *, P < 0.05; **, P < 0.01, ***, P < 0.001; ****, P < 0.0001. (B) Five-fold cross-validation with five repeats were used to evaluate the importance of indicator bacterial genera, including HE versus HP, YE versus YP, HE versus YE, HP versus YP. [file 12866_2023_2857_MOESM1_ESM.pdf]
